# Camptothecin Inhibits Neddylation to Activate the Protective Autophagy Through NF-*κ*B/AMPK/mTOR/ULK1 Axis in Human Esophageal Cancer Cells

**DOI:** 10.3389/fonc.2021.671180

**Published:** 2021-04-08

**Authors:** Yongqing Heng, Yupei Liang, Junqian Zhang, Lihui Li, Wenjuan Zhang, Yanyu Jiang, Shiwen Wang, Lijun Jia

**Affiliations:** ^1^ Cancer Institute, Longhua Hospital, Shanghai University of Traditional Chinese Medicine, Shanghai, China; ^2^ Department of Breast Surgery, Key Laboratory of Breast Cancer in Shanghai, Fudan University Shanghai Cancer Center, Shanghai, China; ^3^ Department of Laboratory Medicine, Huadong Hospital, Affiliated to Fudan University, Shanghai, China

**Keywords:** camptothecin, neddylation, p-I*κ*B*α*, NF-κB/AMPK/mTOR/ULK1, autophagy, apoptosis, esophageal cancer

## Abstract

The neddylation pathway is overactivated in esophageal cancer. Our previous studies indicated that inactivation of neddylation by the NAE inhibitor induced apoptosis and autophagy in cancer cells. Camptothecin (CPT), a well-known anticancer agent, could induce apoptosis and autophagy in cancer cells. However, whether CPT could affect the neddylation pathway and the molecular mechanisms of CPT-induced autophagy in esophageal cancer remains elusive. We found that CPT induced apoptosis and autophagy in esophageal cancer. Mechanistically, CPT inhibited the activity of neddylation and induced the accumulation of p-IkBa to block NF-κB pathway. Furthermore, CPT induced the generation of ROS to modulate the AMPK/mTOR/ULK1 axis to finally promote protective autophagy. In our study, we elucidate a novel mechanism of the NF-*κ*B/AMPK/mTOR/ULK1 pathway in CPT-induced protective autophagy in esophageal cancer cells, which provides a sound rationale for combinational anti-ESCC therapy with CPT and inhibition AMPK/ULK1 pathway.

## Introduction

Post-translational modification of proteins plays crucial roles in the regulation of tumorigenesis and tumor progression. Protein neddylation is an important post-translational modification that conjugates the ubiquitin-like molecule NEDD8 (neuronal precursor cell-expressed developmentally down-regulated protein 8) to substrate proteins (1–4). This process is catalyzed by NEDD8-activating enzyme (NAE, NAE1, and UBA3 heterodimer), transferred to NEDD8-conjugating enzymes E2 and then conjugated to substrate-specific NEDD8-E3 ligases (1–4). The cullin subunits of Cullin-RING E3 ubiquitin ligase (CRL) are the best-characterized substrates of neddylation pathway (5, 6). Accumulated studies show that protein neddylation is elevated in multiple human cancers, and inhibition of this pathway has been developed as a promising anticancer strategy. Mechanistic studies showed that neddylation inhibition effectively induced DNA re-replication stress/DNA damage response, cell cycle arrest, apoptosis, or senescence in a cell-type-dependent manner (7–13). Moreover, neddylation inhibition also induced pro-survival autophagic responses in cancer cells partially *via* modulating the HIF1–REDD1–TSC1 or DEPTOR–mTORC1 pathways (14–16).

Camptothecin (CPT), a topoisomerase I inhibitor, was isolated from the Asian tree Camptotheca acuminate by Wall and Wani in 1966 (17). CPT can form a stable tertiary structure with DNA and topoisomerase I, thus resulting in the formation of the topoisomerase I-CPT complex, which induce DNA double-strand breakage to ultimately promote cell death (18–20). Recent studies have revealed that CPT and its derivatives have significant anticancer efficacy in lung cancer (21), colorectal cancer (22), ovarian cancer (23), and breast cancer (24) *in vitro* and *in vivo*. Mechanistic studies showed that CPT effectively induced cell cycle progression, apoptosis, and other cellular responses (25, 26). For example, CPT induces mitotic arrest through Mad2–Cdc20 complex by activating the JNK-mediated Sp1 pathway (27). In addition, CPT enhanced apoptosis in cancer cells by targeting the 3-UTR regions of Mcl1, Bak1, and p53 through the miR-125b-mediated mitochondrial pathways (20). Furthermore, previous study demonstrated that CPT inhibited the growth and invasion of prostate cancer cells *via* PI3K/AKT, αVβ3/αVβ5 and MMP-2/-9 signaling pathways (28). However, it is completely unknown whether CPT could induce autophagy in esophageal cancer cells.

Autophagy is a process of cellular stress response by which some cytosolic materials are engulfed into autophagosome, followed by lysosome-mediated degradation. Autophagy can be upregulated under different cellular stresses, such as nutrient starvation, ROS accumulation, and reduced cytokine signaling (29, 30). Increasing lines of evidence have confirmed that autophagy is a pro-survival signal in human disease prevention and therapy (31, 32). Targeting the neddylation pathway to inactivate CRL E3 ligases has been shown to induce autophagy (1, 14). In addition, CPT could induce autophagy in some cancer cells. However, the underlying mechanisms of CPT triggering autophagy in ESCC cells remain elusive. Here, for the first time, we reported that neddylation inhibition by CPT significantly induced the accumulation of p-I*κ*B*α* to trigger pro-survival autophagy by modulating NF-*κ*B/AMPK/mTOR/ULK1 axis in esophageal cancer cells, highlighting targeting autophagy as a potential strategy to enhance anti-ESCC therapy of CPT.

## Materials and Methods

### Cell Lines, Culture, and Reagents

Human ESCC cell lines EC1 and EC109 were cultured in Dulbecco’s Modified Eagle’s Medium (Hyclone), containing 10% fetal bovine serum (Biochrom AG) and 1% penicillin–streptomycin solution, at 37°C with 5% carbon dioxide. Chloroquine (CQ), Bafilomycin A1 (BafA1), 3-methyladenine (3MA), and *N*-Acetyl-L-cysteine (NAC) were purchased from Sigma. Compound C (Com. C) was purchased from Selleck. (S)-(+)-camptothecin (CPT, 98%) was purchased from Aladdin Industrial Inc. For *in vitro* studies, CPT stock solution (5 mM) was prepared in dimethyl sulfoxide (DMSO) and stored at −20°C as small aliquots until needed. For *in vivo* studies, CPT was freshly dissolved in 10% 2-hydroxypropyl-b-cyclodextrin (HPBCD) and stored at room temperature before use.

### Cell Viability and Clonogenic Survival Assay

Cells were seeded in 96-well plates (2 × 10^3^ cells/well) and treated with DMSO or CPT. Cell proliferation was determined using the ATPLite Luminescence Assay Kit (PerkinElmer, Waltham, MA, USA) according to manufacturer’s instructions. For the clonogenic assay, 500 cells were seeded in six-well plates and then were treated with DMSO or CPT and cultured for 10 days in six-well plates. The colonies were fixed, stained, and counted under an inverted microscope (Olympus, Tokyo, Japan). Colonies comprising 50 cells or more were counted under an inverted microscope. Three independent experiments were performed.

### Immunoblotting

Cell lysates were prepared for immunoblotting analysis using antibodies against LC3, p62, NEDD8, AMPK, p-AMPK*α* (Thr172), ULK1, p-ULK1 (Ser317), p-H2AX, WEE1, p21, ORC1, Beclin1, ATG5, p-p70S6K (Thr389), p70S6K, 4EBP1, p-4EBP1 (Thr37/46), cleaved PARP, cleaved Caspase-3, I*κ*B*α*, p-I*κ*B*α*, p65, LaminA/C and Tublin (Cell Signaling Technology), Cullin1 (Abcam). ACTIN (Protein Tech) was used as the loading control.

### Gene Silencing Using siRNA

EC1 and EC109 cells were transfected with siRNA oligonucleotides and synthesized by GenePharma (Shanghai, China) using Lipofectamine 2000 (Invitrogen, Carlsbad,CA, USA). The sequences of siRNA are as follows:

siI*κ*B*α*: GCCAGAAATTGCTGAGGCA;
siULK1: CGCCTGTTCTACGAGAAGA;
siBeclin1: CAGTTTGGCACAATCAATA;
siATG5: GGATGAGATAACTGAAAGG.

### Detection of Apoptosis

Cells were treated with CPT at a specified concentration for appointed time. Apoptosis was determined with the Annexin V-FITC/PI Apoptosis Kit (BD Biosciences, San Diego, CA, USA) according to the manufacturer’s instructions.

### Quantification of Reactive Oxygen Species

The quantification of reactive oxygen species (ROS) production was monitored by cell permeable ROS indicator, 2′, 7′-dichlorodihydrofluorescein diacetate (H2-DCFDA) (Sigma). The functional role of ROS generation in autophagy was evaluated by free-radical scavenger NAC (Beyotime). Cells were pre-incubated with 50 μM NAC for 12 h, followed by co-incubation with the indicated chemicals and assessment of autophagy or ROS generation as described above.

### Tumor Formation Assay

For tumor formation assay, five-week-old female athymic nude mice were purchased from the Shanghai Experimental Animal Center (Shanghai, China). 5 × 10^6^ EC1 cells were subcutaneously injected into the right back. Tumor size was measured by a vernier caliper and calculated as (length × width^2^)/2. All procedures were performed in accordance with the National Institutes of Health Guide for the Care and Use of Laboratory Animals.

### Statistical Analysis

The statistical significance of differences between groups was assessed using the Graph Pad Prism 5 software. The unmatched two-tailed t-test was used for the comparison of parameters between two groups. The level of significance was set at *P <*0.05.

## Results

### CPT Induced Autophagy and Suppressed the Growth of Esophageal Cancer Cells *In Vitro* and *In Vivo*

**Figure 1:**
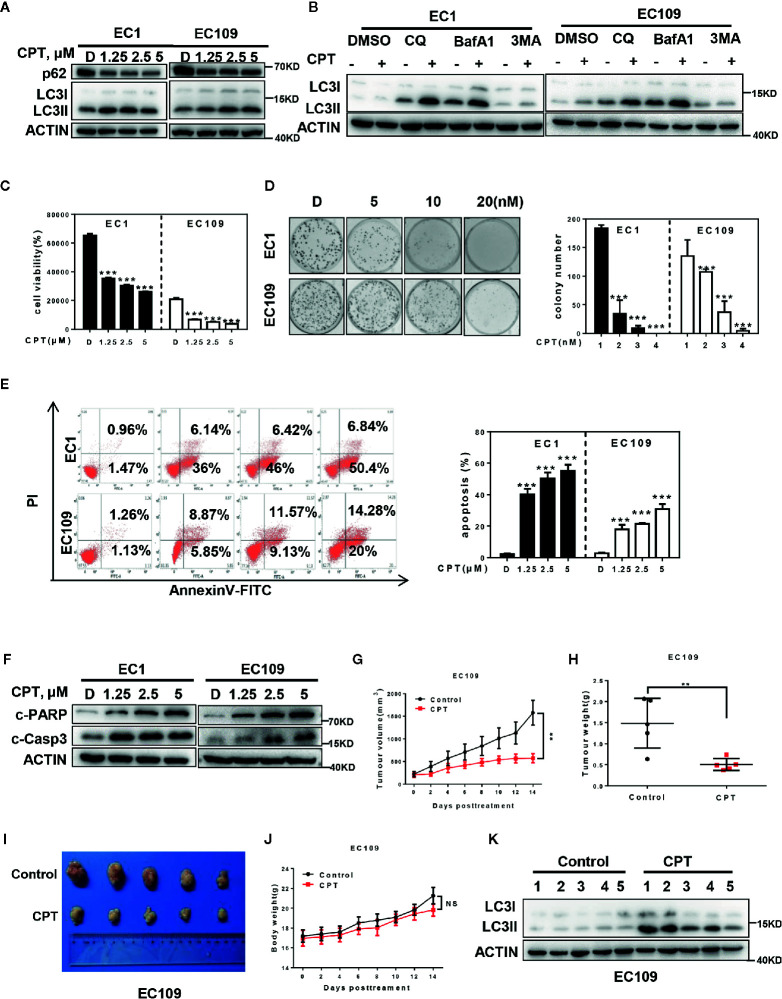
CPT induced autophagy and suppressed the growth of esophageal cancer cells *in vitro* and *in vivo*. **(A)** Cells were treated with the indicated concentrations of CPT for 24 h, and cells were collected and subjected to IB analysis for the expression of LC3 and p62, Actin was used as an equal loading control. **(B)** Autophagic flux analysis. EC1 and EC109 cells treated with DMSO or CPT (2.5 μmol/L) for 24 h were incubated with or without CQ (50 μM), BafA1 (20 nM), or 3MA (5 mM) for 6 h. The treated cells were then collected and subjected to IB analysis with ACTIN as a loading control. **(C)** Cells were treated with the indicated concentrations of CPT for 72 h, and cell viability was assessed by the ATPLite assay (*n* = 4). **(D)** CPT inhibited clonogenic cell survival of ESCC cancer cells. EC1 and EC109 cells were seeded into 60 mm dishes in duplicate and then grown in the presence or absence of CPT for 10 days. The colonies with more than 50 cells were counted, following crystal violet staining (*n* = 3). **(E, F)** CPT induced apoptosis in ESCC cells. **(E)** Cells were treated with the indicated concentrations of CPT for 48 h and subjected to Annexin V-FITC/PI double-staining analysis (*n* = 3). **(F)** Cells were treated with the indicated concentrations of CPT for 24 h, and cell lysates were assessed by IB with specific antibodies against cleaved-Caspase-3 (c-Casp3) and cleaved-PARP (c-PARP). **(G–K)** CPT induced autophagy and suppressed the growth of esophageal cancer cells *in vivo*. Nude mice bearing esophageal cancer xenografts with EC109 cells were administered with CPT at 2.5 mg/kg. The treatments for the nude mice were carried out every 2 days and lasted for 14 days. **(G)** Tumor volumes were determined by caliper measurement, and the data were converted to tumor growth curves. Tumor tissues of mice were collected, photographed, weighed, and stored for further analysis (*n* = 5). **(H)** CPT significantly reduced tumor weight (*n* = 5). **(I)** Images of CPT-treated or control xenograft tumors at the end of experiment. **(J)** No obvious toxicity against body weight was observed during CPT treatment. Body weight of mice was measured twice a week during the treatment (*n* = 5). **(K)** Proteins extracted from tumor tissues were analyzed by IB using anti-LC3. Data were presented as mean ± S.E.M. **P < 0.01 and ***P < 0.001.

To investigate whether CPT could induce autophagy in esophageal cancer cells, we detected the autophagy response after CPT treatment. Firstly, we determined the conversion of LC3-I to LC3-II, a classical marker of autophagy, and found that CPT dramatically induced the conversion of LC3-I to LC3-II and inhibited the expression of p62 in EC1 and EC109 cells (**Figure 1A**). In addition, we performed autophagic flux analysis by treating cells with classical autophagy inhibitors including Chloroquine (CQ), bafilomycin A1 (BafA1), and 3-methyladenine (3MA), respectively. As expected, 3MA inhibited, while BafA1 and CQ enhanced the accumulation of LC3 II, indicating that autophagic flux was intact and supraphysiological autophagic response was induced by CPT treatment (**Figure 1B**). These results convincingly demonstrated that CPT induced autophagy in esophageal cancer cells.

We next evaluated the antitumor activity after CPT treatment in ESCC cells. Firstly, we found that CPT significantly inhibited cell proliferation (**Figure 1C**) and colony formation (**Figure 1D**) in a dose-dependent manner in EC1 and EC109 cells. Next we found that CPT significantly induced apoptosis (**Figures 1E, F** **)**, as best evidenced by the increase of Annexin V-positive cell populations and the accumulation of cleaved-PARP and cleaved-Caspase-3, two classical markers of apoptosis. These results convincingly demonstrated that CPT inhibited cell proliferation and induced apoptosis in esophageal cancer cells.

Having established that CPT induced autophagy and inhibited esophageal cancer cell growth *in vitro*, we next evaluated the antitumor activity and autophagy response after CPT treatment *in vivo*. CPT treatment significantly suppressed tumor growth over time while control tumors grew rapidly, as revealed by size of tumors, tumor growth curve, and tumor weight analysis. CPT-treated tumors progressed slowly, whereas control tumors grew rapidly over time, as shown by tumor growth curve (**Figure 1G**) and tumor weight analysis (**Figure 1H**). Consistently, the size of control tumors was much larger than that of CPT-treated tumors (**Figure 1I**) without obvious treatment-related toxicity, such as body weight loss (**Figure 1J**). In addition, as shown in **Figure 1K**, CPT significantly induced autophagy *in vivo*, as evidenced by the increase of conversion of LC3I to LC3II. Taken together, these findings demonstrated that CPT induced autophagy and inhibited esophageal tumor growth both *in vitro* and *in vivo*.

### CPT-Induced Autophagy Was a Survival Signal in Esophageal Cancer Cells

**Figure 2:**
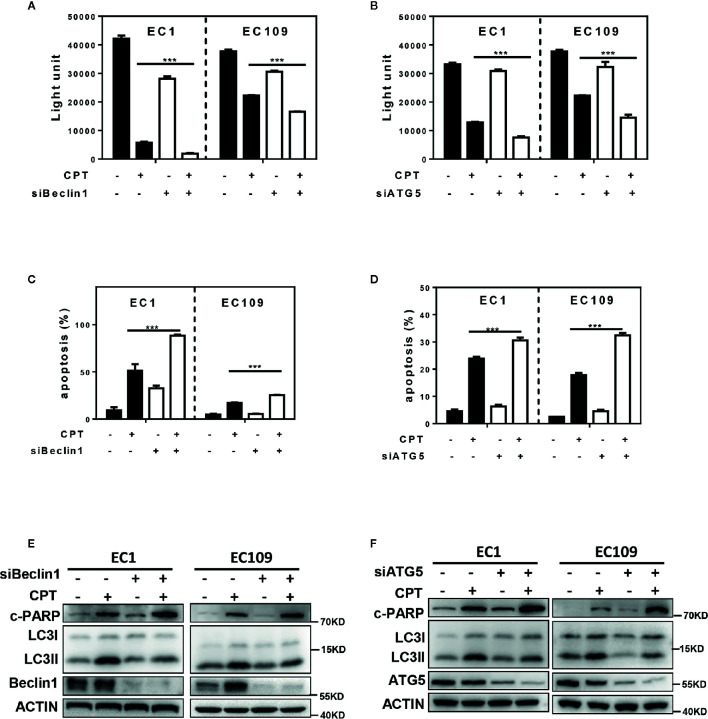
CPT-induced autophagy was a survival signal in esophageal cancer cells. **(A, B)** The proliferation inhibition by CPT treatment was significantly increased by simultaneously blocking autophagy with siBeclin1 or siATG5. The combination of siBeclin1 or siATG5 with CPT in EC1 and EC109 cells significantly increased proliferation inhibition by ATPLite assay (*n* = 3). **(C, D)** Blocking of autophagy pathway by Beclin1 or ATG5 siRNA silencing amplified CPT-induced apoptosis. The combination of siBeclin1 or siATG5 with CPT in EC1 and EC109 cells significantly increased apoptosis by Annexin V-FITC/PI double-staining analysis (*n* = 3). **(E, F)** Beclin1 or ATG5 knockdown increased cleaved PARP expression induced by CPT. Cells were transferred with siRNAs against Beclin 1 **(E)** or ATG5 **(F)** for 48 h, and then treated with CPT at 2.5 μmol/L for 24 h. Knockdown efficiency and cleaved PARP were assessed by IB analysis. Data were presented as mean ± S.E.M. ***P < 0.001.

In order to investigate the role of autophagy response induced by CPT in the growth of ESCC cells, we blocked autophagy pathway *via* siRNA silencing of autophagy essential genes Beclin1 or ATG5 and evaluated its effect on proliferation and apoptosis of esophageal cancer cells. As shown in **Figure 2A**, downregulation of Beclin1 expression effectively enhanced CPT-induced proliferation inhibition in EC1 and EC109 cells. Similarly, downregulation of ATG5 expression effectively enhanced CPT-induced proliferation inhibition in EC1 and EC109 cells (**Figure 2B**). Consistently, the inhibition of autophagic response by siBeclin1 and siATG5 significantly enhanced CPT-induced apoptosis, as best evidenced by the increase of Annexin V-positive cell populations (**Figures 2C, D** **)** and the accumulation of cleaved PARP, a classical marker of apoptosis (**Figures 2E, F** **)** in esophageal cancer cells. These results demonstrated that CPT induced autophagy as a prosurvival signal in esophageal cancer cells.

### AMPK/mTOR/ULK1 Axis Contributes to CPT Induced Autophagy

**Figure 3:**
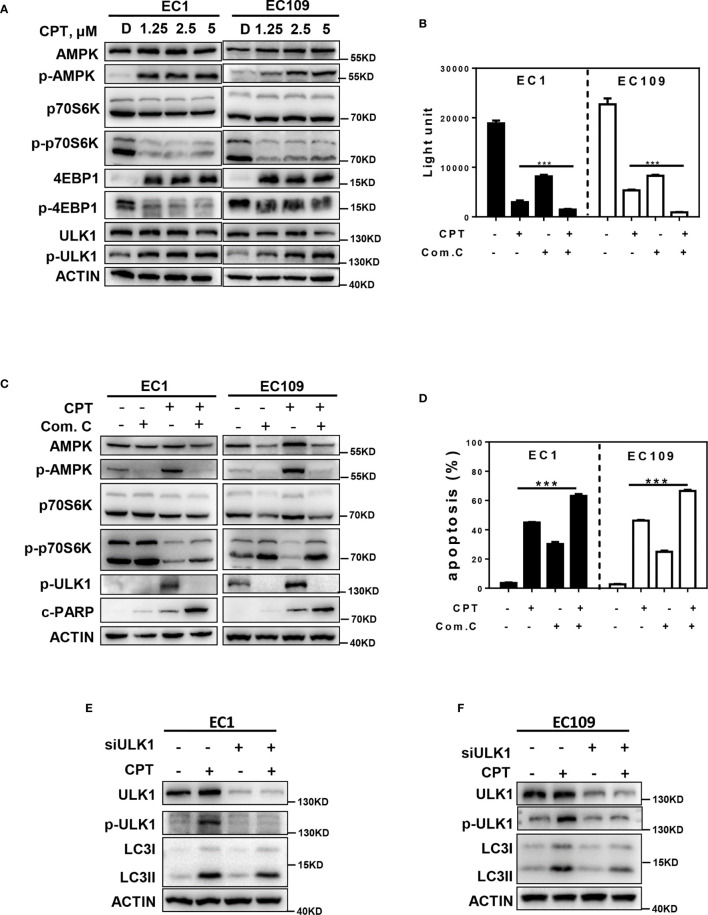
AMPK/mTOR/ULK1 axis contributes to CPT induced autophagy. **(A)** EC1 and EC109 cells were treated with DMSO and 1.25, 2.5, 5 μmol/L CPT for 24 h and then collected and subjected to IB analysis for the expression of AMPK, p-AMPK, p-70S6K, p-p70S6K, 4EBP1, p-4EBP1, ULK1, and p-ULK1. **(B)** EC1 and EC109 cells were treated with 2.5 μmol/L CPT alone or CPT + Com.C (5 μmol/L) for 72 h and subjected to ATPLite assay (*n* = 3). **(C)** EC1 and EC109 cells were treated with 2.5 μmol/L CPT alone or CPT + Com.C (5 μmol/L) for 24 h and subjected to IB analysis for the expression of AMPK, p-AMPK, p-ULK1, p70S6K, p-p70S6K, and c-PARP. **(D)** EC1 and EC109 cells were treated with 2.5 μmol/L CPT alone or CPT + Com.C (5 μmol/L) for 48 h. Apoptosis induction was quantified by Annexin V-FITC/PI double-staining analysis (*n* = 3). **(E, F)** Autophagy was rescued by ULK1 siRNA silencing. ULK1 knockdown largely abrogated CPT-induced conversion of LC3-I to LC3-II in EC1 and EC109 cells. EC1 and EC109 cells were transfected with control or siULK1 for 48 h and then treated with 2.5 μmol/L CPT for 24 h. Knockdown efficiency and LC3 were assessed by IB analysis. Data were presented as mean ± S.E.M. ***P < 0.001.

Previous studies indicated that the activation of AMPK/ULK1 pathway induced autophagy, and inactivation of the mTOR pathway could promote autophagy in multiple human cancers (33). Based on these findings, we determined whether CPT-induced autophagy by modulating the AMPK/mTOR/ULK1 pathway. As shown in **Figure 3A**, we found that CPT activated the AMPK pathway, as best evidenced by the increase of phosphorylation of AMPK and ULK1. In addition, CPT inhibited the mTOR pathway, as best evidenced by the decrease of phosphorylation of p70S6K and 4EBP1. In order to determine the role of AMPK in CPT-induced expression of p-ULK1 and inhibition of p-p70S6K in EC1 and EC109 cells, we used Compound C (an AMPK inhibitor) to inactivate the AMPK pathway and found that inactivation of AMPK significantly reversed CPT-induced expression of p-ULK1 in ESCC cells. Consistently, inactivation of AMPK significantly reversed CPT-inhibited expression of p-p70S6K. Moreover, inactivation of AMPK *via* Compound C treatment significantly increased CPT-induced proliferation inhibition (**Figure 3B**). Additionally, inhibition of AMPK with Compound C significantly enhanced CPT-induced apoptosis, as evidenced by the accumulation of cleaved PARP (**Figure 3C**) and the increase of Annexin V-positive cell populations (**Figure 3D**). In order to determine the role of ULK1 in CPT-induced autophagy in EC1 and EC109 cells, we knockdown ULK1 and found that ULK1 knockdown markedly attenuated the conversion of LC3 I to LC3 II in ESCC cell (**Figures 3E, F** **)**. These findings demonstrated that CPT induced protective autophagy by AMPK/mTOR/ULK1 axis in esophageal cancer cells.

### CPT Induced ROS Generation to Promote Autophagy *via* AMPK/mTOR/ULK1 Axis

**Figure 4:**
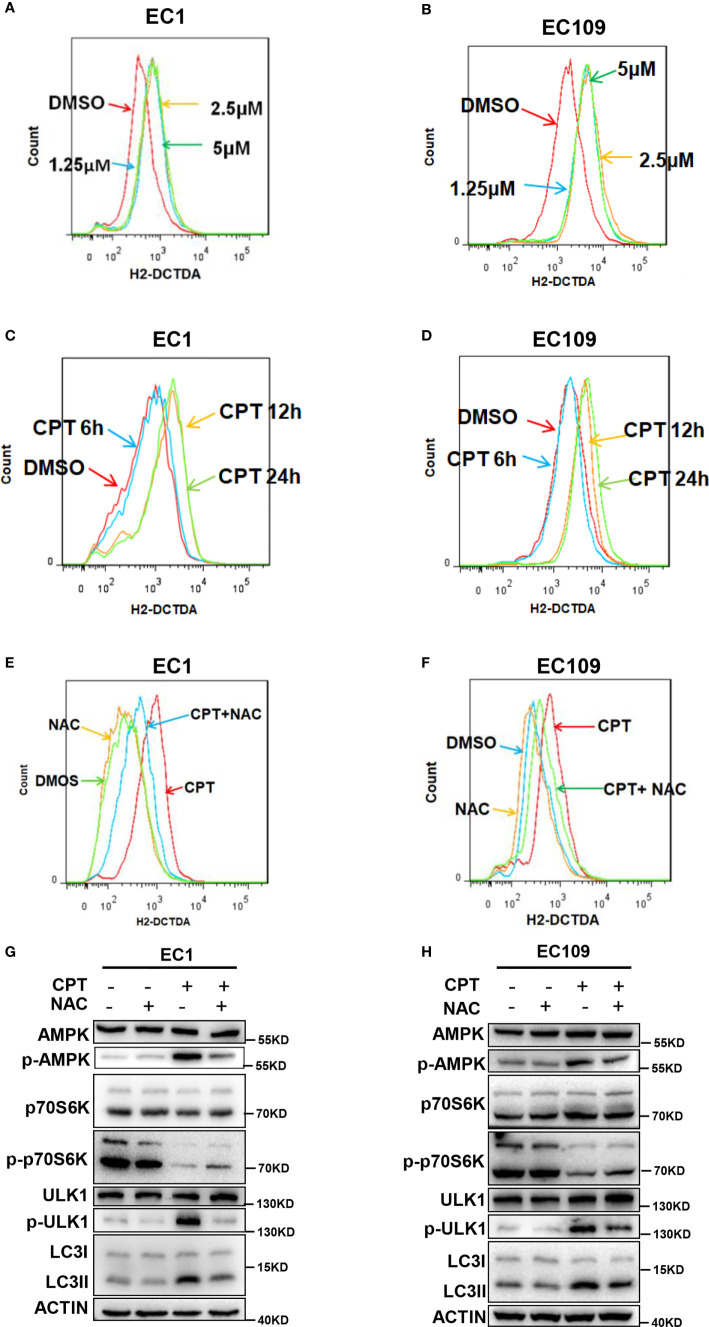
CPT induced ROS generation to promote autophagy *via* AMPK/mTOR/ULK1 axis. **(A–D)** CPT elevated ROS levels in ESCC cells. **(A, B)** Cells were treated with various concentrations of CPT for 24 h. **(C, D)** Cells were treated with 1.25 μmol/L CPT for the indicated time periods. ROS generation was determined by H2-DCFDA staining and flow cytometry. **(E, F)** EC1 and EC109 cells were treated with 1.25 μmol/L CPT alone or CPT + NAC (50 μmol/L) for 12 h and subjected to H2-DCFDA staining analysis for the levels of ROS. **(G, H)** NAC inhibited CPT-induced autophagy and suppressed CPT-modulated AMPK/mTOR/ULK1 axis in ESCC cells. EC1 and EC109 cells were treated with 1.25 μmol/L CPT alone or CPT + NAC (50 μmol/L) for 12 h and subjected to IB analysis for the expression of AMPK, p-AMPK, ULK1, p-ULK1, p70S6K, p-p70S6K, and LC3.

Given that ROS could activate the AMPK pathway to induce autophagy (34–36), we determined whether CPT-induced autophagy was mediated by ROS generation in esophageal cancer cells. We firstly detected cellular ROS level with the cell permeable ROS indicator, 2′, 7-dichlorodihydrofuorescein diacetate (H2-DCFDA), and found that CPT significantly induced ROS production in both EC1 and EC109 cells (**Figures 4A–D**). Furthermore, we determined the role of ROS in CPT-induced AMPK/ULK1 pathway and CPT-inhibited mTOR pathway. We used NAC, a classical ROS scavenger, and found that NAC prevented CPT induced the generation of ROS (**Figures 4E, F** **)** and found that ROS reduction markedly attenuated CPT-induced the expression of p-AMPK, p-ULK1, LC3II and CPT-inhibited the expression of p-p70s6k (**Figures 4G, H** **)**. Based on these observations, we concluded that CPT-induced ROS production modulated the AMPK/mTOR/ULK1 pathway to induce autophagy in esophageal cancer cells.

### ROS-Mediated Autophagy Is Attributed to p-IκBα Accumulation by Neddylation Inactivation

**Figure 5:**
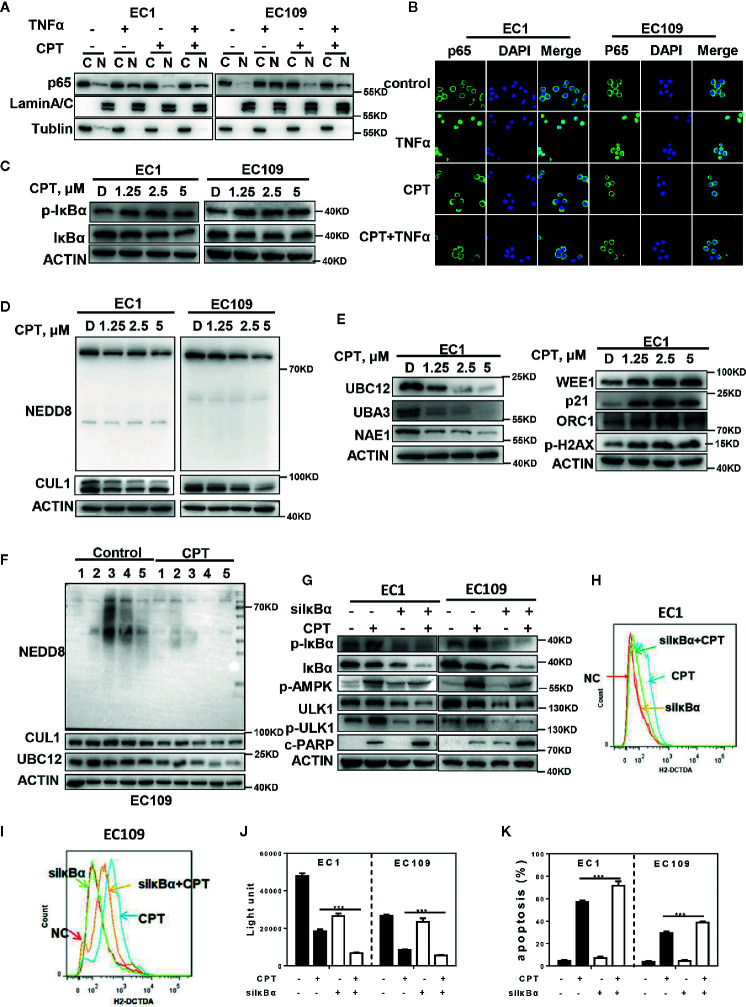
ROS-mediated autophagy is attributed to p- IκBα accumulation by neddylation inactivation. **(A–C)** CPT inhibited the activation of NF-*κ*B pathway. **(A, B)** CPT prevented p65 NF-*κ*B translocation to the nucleus induced by TNFα. ESCC cells were cultured in the presence or absence of 2.5 μmol/L CPT (12 h) and stimulated concurrently with TNFα (100 ng/ml) for 30 min. **(A)** p65 isoform of NF-*κ*B was determined by western blot analysis using nuclear (N) and cytosolic (C) fractions of ESCC cells treated as indicated. Lamin A/C and Tubulin were analyzed to demonstrate the presence of nuclear and cytosolic fractions, respectively. **(B)** p65 NF-*κ*B subcellular localization was determined by immunofluorescence staining for endogenous p65 NF-*κ*B (green). **(C)** EC1 and EC109 cells were treated with 2.5 μmol/L CPT for 24 h and cell lysates were assessed by IB with specific antibody against I*κ*B*α* and p-I*κ*B*α*. **(D, E)** CPT inhibited neddylation modification. **(D)** Immunoblotting was used to analyze the neddylation levels of cullin1 and global protein neddylation upon CPT treatment for 24 h with various concentrations. **(E)** ESCC cells were treated with CPT (0, 1.25, 2.5, and 5 μmol/L) for 24 h, followed by IB analysis using antibodies against NAE1, UBA3, UBC12, WEE1, p21, ORC1, p-H2AX, ACTIN as a loading control. **(F)** CPT inhibited neddylation pathway *in vivo*. Nude mice bearing esophageal cancer xenografts with EC109 cells were administered with CPT at 2.5 mg/kg. The treatments for the nude mice were carried out every 2 days and lasted for 14 days. Proteins extracted from tumor tissues were analyzed by IB using anti-NEDD8, cullin1, and UBC12. **(G, K)** ESCC cells were transfected with I*κ*B*α* siRNA, then treated with 2.5 μmol/L CPT for 48 h. p-AMPK, p-ULK1, cleaved PARP activity were assessed by IB analysis **(G)**. ROS generation was determined by H2-DCFDA staining and flow cytometry **(H, I)**. Cell viability was measured using the ATPLite assay **(J)** and apoptosis was detected by annexin V and PI double staining **(K)** (*n* = 3). Data were presented as mean ± S.E.M. ***P < 0.001.

Since the inactivation of NF-*κ*B could induce ROS generation (37, 38), we next determined whether ROS/AMPK/mTOR/ULK1 axis-induced autophagy is mediated by the NF-*κ*B pathway. Firstly, we found that pretreating cells with CPT prior to TNFα (an activator of NF-*κ*B) stimulation significantly inhibited protein level of p65 NF-*κ*B in the nuclear fraction of esophageal cancer cells, suggesting that CPT inhibited the activation of NF-*κ*B pathway (**Figure 5A**). Furthermore, immunofluorescence staining demonstrated that cells stimulated with TNFα showed prominent p65 NF-*κ*B accumulation in the nucleus (**Figure 5B**). Translocation of NF-*κ*B to the nucleus is allowed by the phosphorylation of I*κ*B*α*, resulting in its ubiquitination and degradation by CRL complex. Based on this, we hypothesized that CPT may induce p-I*κ*B*α* accumulation due to the inactivation of CRL E3 ligase, and therefore activate ROS-mediated AMPK/mTOR/ULK1 axis to activate autophagy. As shown in **Figure 5C**, CPT significantly induced the expression of p-I*κ*B*α* in both EC1 and EC109 cells. Interestingly, we found that CPT indeed suppressed the global protein neddylation and the neddylation levels of Cullin1 (**Figure 5D**). We further explored the mechanism of CPT-induced neddylation pathway in esophageal cancer cells. The key neddylation enzymes, NAE1, UBA3 and UBC12, were obviously suppressed upon CPT treatment in EC1 cells (**Figure 5E**). Furthermore, CRL substrates, including WEE1, p21, ORC1, and p-H2AX, were accumulated upon CPT treatment (**Figure 5E**). Having established that CPT inhibited neddylation pathway *in vitro*, we next evaluated whether CPT inactivated neddylation after CPT treatment *in vivo*. As shown in **Figure 5F**, CPT indeed suppressed the global protein neddylation, cullin1 neddylation, and the expression of the neddylation enzyme UBC12. These findings demonstrated that CPT inhibited the protein neddylation pathway *in vitro* and *in vivo*.

To further investigate the potential role of I*κ*B*α* in CPT-induced ROS production and autophagy, we downregulated the I*κ*B*α* expression in esophageal cancer cells. We found that I*κ*B*α* knockdown markedly attenuated CPT-induced expression of p-AMPK, p-ULK1 (**Figure 5G**) and the generation of ROS (**Figures 5H, I** **)**. Furthermore, we found that I*κ*B*α* knockdown significantly enhanced CPT-induced proliferation inhibition (**Figure 5J**). In addition, I*κ*B*α* knockdown significantly enhanced CPT-induced apoptosis, as evidenced by the accumulation of cleaved PARP (**Figure 5G**) and the increase of Annexin V-positive cell populations (**Figure 5K**). These findings collectively demonstrated that CPT inhibited NF-*κ*B pathway to promote ROS generation, which modulated the AMPK/mTOR/ULK1 axis to eventually induce autophagy in esophageal cancer cells.

## Discussion

Esophageal cancer is one of the most human malignant tumors with high recurrence rate and poor long-term survival (39, 40). The severe threat of esophageal cancer to human health raises an urgent necessity to further elucidate the mechanisms for esophageal carcinogenesis and need novel effective therapeutic strategies. Recently, protein neddylation pathway has emerged as a potential anti-ESCC target, as supported by the discovery of overactivation of the neddylation pathway in esophageal cancer. Our present work demonstrated for the first time that CPT inhibited cullin neddylation, inactivated CRLs and induced the accumulation of classical CRL substrates p-I*κ*B*α*. Mechanistic investigations further revealed that the neddylation inhibition by CPT induced the generation of ROS to modulate AMPK/mTOR/ULK1 axis to induce autophagy in esophageal cancer cells. Therefore, the neddylation pathway may serve as an important drug target for CPT to mediate cell death in ESCC cells.

Recently, the neddylation pathway, including its three enzymes NAE, UBC12 and NEDD8, has been reported to be overactivated in many kinds of cancer cells, indicating the neddylation pathway as a promising anticancer target (8, 9, 41–43). In our study, we discovered for the first time that CPT inhibited cullin neddylation to inactivate CRLs, as evidenced by the accumulation of CRLs substrate p-I*κ*B*α*. Furthermore, we found that CPT reduced the expression of NAE1, UBA3, and BUC12. However, it is unclear how neddylation enzymes are downregulated by CPT in esophageal cancer. These findings establish the necessity to explore the mechanism by which CPT inhibits neddylation in future studies.

AMPK is an important cellular energy sensor and acts as a duplex molecule in cancer development and progression. In the early phase, AMPK may function as a tumor suppressor and its activation would lead to cell cycle arrest and tumor growth inhibition, thus playing a critical role in cancer prevention (44–47). However, it should be noted that AMPK might protect tumor cells from death-inducing events by maintaining intracellular homeostasis, once the tumors are established and finally lead to cancer drug resistance and metastasis (45, 48). For example, AMPK-deficient tumor cells were more susceptible to cell death induced by glucose deprivation, suggesting that AMPK activation is a pro-survival signal in cancer cells (49). In our study, we illustrated that CPT treatment induced AMPK activation to trigger autophagic response as a pro-survival signal in esophageal cancer cells, which provide a potential combination strategy of dually targeting AMPK and neddylation pathway for effective anti-ESCC therapy.

**Figure 6:**
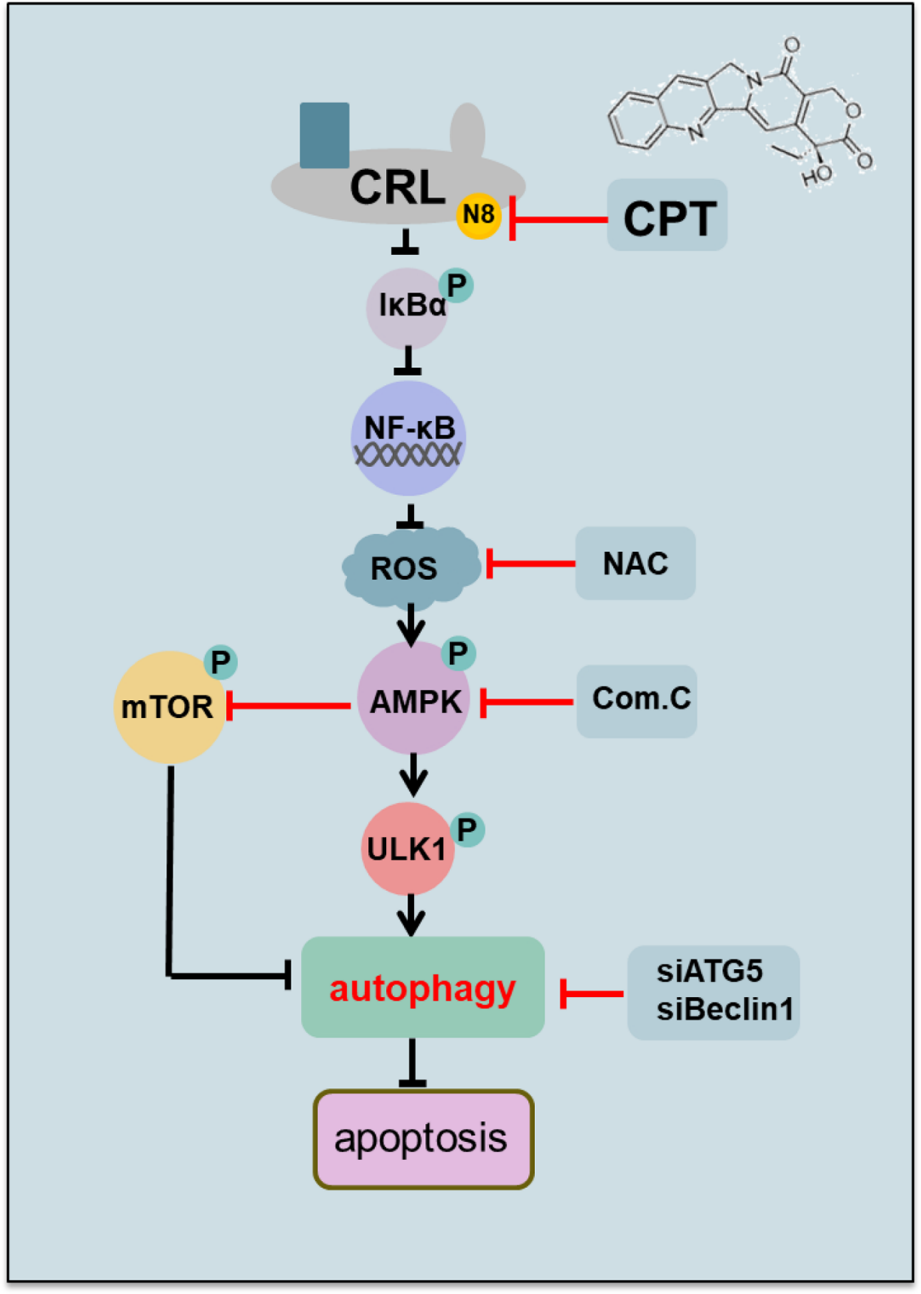
Working model. CPT inhibited cullin neddylation, inactivated CRLs and induced the accumulation of classical CRL substrates p-I*κ*B*α*. Mechanistic investigations further revealed that the neddylation inhibition by CPT induced the generation of ROS to modulate AMPK/mTOR/ULK1 axis to induce autophagy in esophageal cancer cells.

Our study suggested the following working model (**Figure 6**). We first time found that CPT promote autophagy in esophageal cancer cells. Mechanistically, CPT inactivates neddylation pathway, which induce the expression of p-I*κ*B*α* to modulate AMPK/mTOR/ULK1 pathway to trigger pro-survival autophagy, whereas targeting this pathway blocks the autophagic response and thus sensitizes cancer cells to CPT-induced apoptosis. These findings provide a potential combination strategy of dually targeting AMPK/mTOR/ULK1 axis and neddylation pathway for effective anti-ESCC therapy.

## Data Availability Statement

The original contributions presented in the study are included in the article/supplementary material. Further inquiries can be directed to the corresponding author.

## Ethics Statement

The animal study was reviewed and approved by Animal Experimental Ethics Committee of Shanghai University of Traditional Chinese Medicine.

## Author Contributions

YH, YL, and LJ conceived the general framework of this study and designed the experiments. YH, YL, JZ, and LL performed the experiments. WZ, YJ, and SW provided technical or material support. YH and YL prepared the manuscript. LJ supervised this study. All authors contributed to the article and approved the submitted version.

## Funding

This work was supported by the following funds: The Chinese Minister of Science and Technology grant (2016YFA0501800), National Natural Science Foundation of China (Grants 81625018, 81820108022, 82002973), Program of Shanghai Academic/Technology Research Leader (18XD1403800), Innovation Program of Shanghai Municipal Education Commission (2019-01-07-00-10-E00056), and National Thirteenth Five-Year Science and Technology Major Special Project for New Drug and Development (2017ZX09304001).

## Conflict of Interest

The authors declare that the research was conducted in the absence of any commercial or financial relationships that could be construed as a potential conflict of interest.

## Abbreviations

NEDD8, neural precursor cell expressed developmentally down-regulated 8; CRL, Cullin-RING E3 ligase; ESCC, Esophageal squamous cell carcinoma; CQ, chloroquine; BafA1, Bafilomycin A1; 3MA, 3-methyladenine; IB, immunoblotting; Com.C, Compound C.

## References

[1] Zhou L Zhang W Sun Y Jia L. Protein neddylation and its alterations in human cancers for targeted therapy. Cell Signal (2018) 44:92–102. 10.1016/j.cellsig.2018.01.009 29331584 PMC5829022

[2] Soucy TA Dick LR Smith PG Milhollen MA Brownell JE. The NEDD8 Conjugation Pathway and Its Relevance in Cancer Biology and Therapy. Genes Cancer (2010) 1(7):708–16. 10.1177/1947601910382898 PMC3092238 21779466

[3] Duncan K Schafer G Vava A Parker MI Zerbini LF. Targeting neddylation in cancer therapy. Future Oncol (2012) 8(11):1461–70. 10.2217/fon.12.131 23148618

[4] Xirodimas DP. Novel substrates and functions for the ubiquitin-like molecule NEDD8. Biochem Soc Trans (2008) 36(Pt 5):802–6. 10.1042/BST0360802 18793140

[5] Petroski MD Deshaies RJ. Function and regulation of cullin-RING ubiquitin ligases. Nat Rev Mol Cell Biol (2005) 6(1):9–20. 10.1038/nrm1547 15688063

[6] Deshaies RJ Joazeiro CA. RING domain E3 ubiquitin ligases. Annu Rev Biochem (2009) 78:399–434. 10.1146/annurev.biochem.78.101807.093809 19489725

[7] Godbersen JC Humphries LA Danilova OV Kebbekus PE Brown JR Eastman A. Correction: The Nedd8-Activating Enzyme Inhibitor MLN4924 Thwarts Microenvironment-Driven NF-kappaB Activation and Induces Apoptosis in Chronic Lymphocytic Leukemia B Cells. Clin Cancer Res (2016) 22(16):4274. 10.1158/1078-0432.CCR-16-1475 24634471 PMC3960291

[8] Chen P Hu T Liang Y Li P Chen X Zhang J. Neddylation Inhibition Activates the Extrinsic Apoptosis Pathway through ATF4-CHOP-DR5 Axis in Human Esophageal Cancer Cells. Clin Cancer Res (2016) 22(16):4145–57. 10.1158/1078-0432.CCR-15-2254 26983464

[9] Li L Wang M Yu G Chen P Li H Wei D. Overactivated neddylation pathway as a therapeutic target in lung cancer. J Natl Cancer Inst (2014) 106(6):dju083. 10.1093/jnci/dju083 24853380

[10] Lin JJ Milhollen MA Smith PG Narayanan U Dutta A. NEDD8-targeting drug MLN4924 elicits DNA rereplication by stabilizing Cdt1 in S phase, triggering checkpoint activation, apoptosis, and senescence in cancer cells. Cancer Res (2010) 70(24):10310–20. 10.1158/0008-5472.CAN-10-2062 PMC3059213 21159650

[11] Milhollen MA Narayanan U Soucy TA Veiby PO Smith PG Amidon B. Inhibition of NEDD8-activating enzyme induces rereplication and apoptosis in human tumor cells consistent with deregulating CDT1 turnover. Cancer Res (2011) 71(8):3042–51. 10.1158/0008-5472.CAN-10-2122 21487042

[12] Soucy TA Smith PG Milhollen MA Berger AJ Gavin JM Adhikari S. An inhibitor of NEDD8-activating enzyme as a new approach to treat cancer. Nature (2009) 458(7239):732–6. 10.1038/nature07884 19360080

[13] Zhao Y Morgan MA Sun Y. Targeting Neddylation pathways to inactivate cullin-RING ligases for anticancer therapy. Antioxid Redox Signal (2014) 21(17):2383–400. 10.1089/ars.2013.5795 PMC4241876 24410571

[14] Zhao Y Xiong X Jia L Sun Y. Targeting Cullin-RING ligases by MLN4924 induces autophagy via modulating the HIF1-REDD1-TSC1-mTORC1-DEPTOR axis. Cell Death Dis (2012) 3:e386. 10.1038/cddis.2012.125 22951983 PMC3461362

[15] Zhao Y Sun Y. Targeting the mTOR-DEPTOR pathway by CRL E3 ubiquitin ligases: therapeutic application. Neoplasia (2012) 14(5):360–7. 10.1593/neo.12532 PMC3384423 22745582

[16] Yang D Zhao Y Liu J Sun Y Jia L. Protective autophagy induced by RBX1/ROC1 knockdown or CRL inactivation via modulating the DEPTOR-MTOR axis. Autophagy (2012) 8(12):1856–8. 10.4161/auto.22024 PMC3541304 22965024

[17] Wall ME Wani MC Cook CE Palmer KH Mcphail AT Sim G. Plant Antitumor Agents. I. The Isolation and Structure of Camptothecin, a Novel Alkaloidal Leukemia and Tumor Inhibitor from Camptotheca acuminata1,2. J Am Chem Soc (1966) 88(16):3888–90. 10.1021/ja00968a057

[18] Eng WK Faucette L Johnson RK Sternglanz R. Evidence that DNA topoisomerase I is necessary for the cytotoxic effects of camptothecin. Mol Pharmacol (1988) 34(6):755–60. 2849043

[19] Wadkins RM Bearss D Manikumar G Wani MC Wall ME Von Hoff DD. Topoisomerase I-DNA complex stability induced by camptothecins and its role in drug activity. Curr Med Chem Anticancer Agents (2004) 4(4):327–34. 10.2174/1568011043352894 15281905

[20] Zeng CW Zhang XJ Lin KY Ye H Feng SY Zhang H. Camptothecin induces apoptosis in cancer cells via microRNA-125b-mediated mitochondrial pathways. Mol Pharmacol (2012) 81(4):578–86. 10.1124/mol.111.076794 22252650

[21] Chiu YH Hsu SH Hsu HW Huang KC Liu W Wu CY. Human nonsmall cell lung cancer cells can be sensitized to camptothecin by modulating autophagy. Int J Oncol (2018) 53(5):1967–79. 10.3892/ijo.2018.4523 PMC6192723 30106130

[22] Arakawa Y Ozaki K Okawa Y Yamada H. Three missense mutations of DNA topoisomerase I in highly camptothecin-resistant colon cancer cell sublines. Oncol Rep (2013) 30(3):1053–8. 10.3892/or.2013.2594 PMC3783056 23836376

[23] Shaikh IM Tan KB Chaudhury A Liu Y Tan BJ Tan BM. Liposome co-encapsulation of synergistic combination of irinotecan and doxorubicin for the treatment of intraperitoneally grown ovarian tumor xenograft. J Control Release (2013) 172(3):852–61. 10.1016/j.jconrel.2013.10.025 24459693

[24] Landgraf M Lahr CA Kaur I Shafiee A Sanchez-Herrero A Janowicz PW. Targeted camptothecin delivery via silicon nanoparticles reduces breast cancer metastasis. Biomaterials (2020) 240:119791. 10.1016/j.biomaterials.2020.119791 32109589

[25] Prasad Tharanga Jayasooriya RG Dilshara MG Neelaka Molagoda IM Park C Park SR Lee S. Camptothecin induces G2/M phase arrest through the ATM-Chk2-Cdc25C axis as a result of autophagy-induced cytoprotection: Implications of reactive oxygen species. Oncotarget (2018) 9(31):21744–57. 10.18632/oncotarget.24934 PMC5955160 29774099

[26] Yin X Sun H Yu D Liang Y Yuan Z Ge Y. Hydroxycamptothecin induces apoptosis of human tenon’s capsule fibroblasts by activating the PERK signaling pathway. Invest Ophthalmol Vis Sci (2013) 54(7):4749–58. 10.1167/iovs.12-11447 23761079

[27] Dilshara MG Jayasooriya R Karunarathne W Choi YH Kim GY. Camptothecin induces mitotic arrest through Mad2-Cdc20 complex by activating the JNK-mediated Sp1 pathway. Food Chem Toxicol (2019) 127:143–55. 10.1016/j.fct.2019.03.026 30885713

[28] Sun LC Luo J Mackey LV Fuselier JA Coy DH. A conjugate of camptothecin and a somatostatin analog against prostate cancer cell invasion via a possible signaling pathway involving PI3K/Akt, alphaVbeta3/alphaVbeta5 and MMP-2/-9. Cancer Lett (2007) 246(1-2):157–66. 10.1016/j.canlet.2006.02.016 16644105

[29] Czarny P Pawlowska E Bialkowska-Warzecha J Kaarniranta K Blasiak J. Autophagy in DNA Damage Response. Int J Mol Sci (2015) 16(2):2641–62. 10.3390/ijms16022641 PMC4346856 25625517

[30] Song X Narzt MS Nagelreiter IM Hohensinner P Terlecki-Zaniewicz L Tschachler E. Autophagy deficient keratinocytes display increased DNA damage, senescence and aberrant lipid composition after oxidative stress in vitro and in vivo. Redox Biol (2017) 11:219–30. 10.1016/j.redox.2016.12.015 PMC5192251 28012437

[31] Deng S Shanmugam MK Kumar AP Yap CT Sethi G Bishayee A. Targeting autophagy using natural compounds for cancer prevention and therapy. Cancer (2019) 125(8):1228–46. 10.1002/cncr.31978 30748003

[32] Galluzzi L Bravo-San Pedro JM Levine B Green DR Kroemer G. Pharmacological modulation of autophagy: therapeutic potential and persisting obstacles. Nat Rev Drug Discovery (2017) 16(7):487–511. 10.1038/nrd.2017.22 28529316 PMC5713640

[33] Kim J Kundu M Viollet B Guan KL. AMPK and mTOR regulate autophagy through direct phosphorylation of Ulk1. Nat Cell Biol (2011) 13(2):132–41. 10.1038/ncb2152 PMC3987946 21258367

[34] Dewaele M Maes H Agostinis P. ROS-mediated mechanisms of autophagy stimulation and their relevance in cancer therapy. Autophagy (2014) 6(7):838–54. 10.4161/auto.6.7.12113 20505317

[35] Russell RC Yuan H-X Guan K-L. Autophagy regulation by nutrient signaling. Cell Res (2013) 24(1):42–57. 10.1038/cr.2013.166 24343578 PMC3879708

[36] Rabinovitch RC Samborska B Faubert B Ma EH Gravel SP Andrzejewski S. AMPK Maintains Cellular Metabolic Homeostasis through Regulation of Mitochondrial Reactive Oxygen Species. Cell Rep (2017) 21(1):1–9. 10.1016/j.celrep.2017.09.026 28978464

[37] Morgan MJ Liu Z-g. Crosstalk of reactive oxygen species and NF-κB signaling. Cell Res (2010) 21(1):103–15. 10.1038/cr.2010.178 PMC3193400 21187859

[38] Nakajima S Kitamura M. Bidirectional regulation of NF-κB by reactive oxygen species: A role of unfolded protein response. Free Radical Biol Med (2013) 65:162–74. 10.1016/j.freeradbiomed.2013.06.020 23792277

[39] Bray F Ferlay J Soerjomataram I Siegel RL Torre LA Jemal A. Global cancer statistics 2018: GLOBOCAN estimates of incidence and mortality worldwide for 36 cancers in 185 countries. CA: A Cancer J Clin (2018) 68(6):394–424. 10.3322/caac.21492 30207593

[40] Lagergren J Smyth E Cunningham D Lagergren P. Oesophageal cancer. Lancet (2017) 390(10110):2383–96. 10.1016/S0140-6736(17)31462-9 28648400

[41] Gao Q Yu GY Shi JY Li LH Zhang WJ Wang ZC. Neddylation pathway is up-regulated in human intrahepatic cholangiocarcinoma and serves as a potential therapeutic target. Oncotarget (2014) 5(17):7820–32. 10.18632/oncotarget.2309 PMC4202163 25229838

[42] Hua W Li C Yang Z Li L Jiang Y Yu G. Suppression of glioblastoma by targeting the overactivated protein neddylation pathway. Neuro Oncol (2015) 17(10):1333–43. 10.1093/neuonc/nov066 PMC4578582 25904638

[43] Xie P Zhang M He S Lu K Chen Y Xing G. The covalent modifier Nedd8 is critical for the activation of Smurf1 ubiquitin ligase in tumorigenesis. Nat Commun (2014) 5:3733. 10.1038/ncomms4733 24821572

[44] Mihaylova MM Shaw RJ. The AMPK signalling pathway coordinates cell growth, autophagy and metabolism. Nat Cell Biol (2011) 13(9):1016–23. 10.1038/ncb2329 PMC3249400 21892142

[45] Kim J Yang G Kim Y Kim J Ha J. AMPK activators: mechanisms of action and physiological activities. Exp Mol Med (2016) 48(4):e224–e. 10.1038/emm.2016.16 PMC4855276 27034026

[46] Shackelford DB Shaw RJ. The LKB1-AMPK pathway: metabolism and growth control in tumour suppression. Nat Rev Cancer (2009) 9(8):563–75. 10.1038/nrc2676 PMC2756045 19629071

[47] Jones RG Plas DR Kubek S Buzzai M Mu J Xu Y. AMP-activated protein kinase induces a p53-dependent metabolic checkpoint. Mol Cell (2005) 18(3):283–93. 10.1016/j.molcel.2005.03.027 15866171

[48] Vara-Ciruelos D Russell FM Hardie DG. The strange case of AMPK and cancer: Dr Jekyll or Mr Hyde? (dagger) Open Biol (2019) 9(7):190099. 10.1098/rsob.190099 31288625 PMC6685927

[49] Jeon S-M Chandel NS Hay N. AMPK regulates NADPH homeostasis to promote tumour cell survival during energy stress. Nature (2012) 485(7400):661–5. 10.1038/nature11066 PMC3607316 22660331

